# Effects of Two Different Methods of Teeth Grinding on Dental Injuries, Skin Lesions, Growth and Behaviour of Suckling Piglets Compared to a Non-Treated Control Group

**DOI:** 10.3390/ani14091318

**Published:** 2024-04-28

**Authors:** Carolin Bernarda Timphaus, Franziska Anna kleine Kruthaup, Fritjof Freise, Swetlana Herbrandt, Elisabeth große Beilage, Michaela Fels

**Affiliations:** 1 Field Station for Epidemiology, University of Veterinary Medicine, Hannover Foundation, Büscheler Straße 9, D-49456 Bakum, Germanyelisabeth.grosse.beilage@tiho-hannover.de (E.g.B.); 2 Institute for Animal Hygiene, Animal Welfare and Farm Animal Behaviour, University of Veterinary Medicine, Hannover Foundation, Bischofsholer Damm 15, D-30173 Hannover, Germany; 3 Institute for Biometry, Epidemiology and Information Processing, University of Veterinary Medicine, Hannover Foundation, Bünteweg 2, D-30559 Hannover, Germany; fritjof.freise@tiho-hannover.de; 4 Statistical Consulting and Analysis, Center for Higher Education, TU Dortmund University, Vogelpothsweg 78, D-44227 Dortmund, Germany; swetlana.herbrandt@tu-dortmund.de

**Keywords:** pig, teeth grinding, teacup grinding head, roller grinding head, behaviour, animal welfare

## Abstract

**Simple Summary:**

In suckling piglets, the third incisors and canines are already formed at birth. This can lead to bite injuries when suckling on the sow’s teats and during hierarchy formation among littermates. For this reason, some farms routinely grind the teeth with a rotating grinding head. However, grinding can lead to tooth injuries, inflammation and pain. The aim of this study was to compare the common roller grinding head with a so-called teacup grinding head (a teacup-shaped attachment that is supposed to adapt better to the individual tooth) and with a control group that was not ground at all. Therefore, dental injuries, weight gain, skin lesions, mortality and behaviour of piglets were determined. The teacup method caused significantly fewer opened tooth pulps than the roller head method. Nonetheless, no difference was found with regard to growth, lesions, mortality and behaviour when comparing the two grinding methods and the control group. It can be concluded that the teacup method is a more animal-friendly option. Nevertheless, it must be considered individually for each farm whether there is a need for grinding at all. If grinding cannot be dispensed with, the use of the teacup method is recommended in the interest of animal welfare.

**Abstract:**

Teeth grinding in suckling piglets is performed on many farms to protect the piglets’ littermates and the sow’s udder from injuries caused by the piglets’ canines and third incisors. In this study, the effects of two teeth-grinding methods on the piglets’ dental health and welfare were investigated. The piglets of a litter were evenly assigned to a treatment: one-third of littermates were ground with a roller grinding head (RG), one-third with a teacup grinding head (TCG), and one-third were not ground at all (CG). A random sample of 100 animals each from the RG and TCG treatment was examined for tooth injuries, i.e., dental pulp openings. Additionally, behavioural analysis was performed (*n* = 650 piglets), and skin lesions, growth and mortality were determined (*n* = 1565 piglets). TCG piglets had a lower risk (*p* < 0.001) of pulp opening than RG piglets (0.08 ± 0.31 vs. 2.67 ± 1.67 opened pulps per piglet). Mortality, growth, skin lesions and behaviour of piglets were not influenced by treatment (*p* > 0.05). This study showed that both teeth-grinding methods led to pulp openings. If teeth grinding cannot be avoided on a farm, using the teacup grinding head is recommended concerning animal health and welfare.

## 1. Introduction

Increased consumer expectations regarding animal welfare pose new challenges for agriculture [1]. There are a number of welfare issues in pig farming that are currently being addressed, such as tail docking or housing of sows in farrowing crates. Teeth grinding in suckling piglets, which is performed on many farms, can also impair welfare [2] but still receives less attention in scientific research.

In newborn piglets, the canines and third incisors are already pronounced at birth [3]. When piglets are fighting to establish a suckling order at the sow, injuries are caused by the small, pointed teeth—often on the piglets’ faces [4]. Furthermore, the already-pronounced teeth often injure the sow’s udder [5]. Therefore, grinding of the eight teeth already pronounced at birth is often one of the zootechnical measures in piglet production, as well as ear tagging, castration, and tail docking [6]. It was shown that teeth resection led to a significant reduction in injuries to the faces of piglets and teats of sows [5].

In accordance with European legislation [7] and the German Animal Protection Act [8], teeth resection may only be performed under certain conditions. It must be proven to be essential for the protection of the sow and littermates, and it may only be performed on animals up to eight days old. This resection may be performed without analgesia and anaesthesia.

In the past, the teeth of piglets were simply cut off using a so-called side cutter. This is now prohibited in accordance with German animal welfare legislation, as it has often led to high-grade inflammation of the tooth socket and pulp [3]. The teeth should only be ground off with special equipment. A study has shown that a partial resection (only the distal third of the tooth) or a complete resection of the teeth (with the exception of the gums) led to significantly fewer lesions compared to intact teeth [9]. All animals in the litter, or only some of them, can be tooth ground, whereby the teeth of the lightest piglets are spared. This has the advantage that piglets with low birth weights remain competitive with the heavier piglets. [10]. A common problem when grinding the teeth using a standard grinding head is the opening of the dental pulp. The open pulp causes pain and can be an entry for bacteria and other pathogens. The rotating head of the standard grinder does not require much pressure to damage the 1.3-millimetre-thick occlusal layer and expose the pulp [11]. In addition, injuries to the oral mucosa can occur, which often result in inflammation of the gingiva [12]. Fractured and chipped teeth are also not uncommon when using standard grinders [13]. There is an alternative to the standard grinding head, which is supposed to be a more animal-friendly method. The special feature of the grinding head—called Teacup—is the concave grinding surface, which adapts better to the tooth during grinding [14]. Currently, the best alternative would be to dispense with grinding in general. However, this could also have negative effects on the health of the sows and piglets since injuries can be caused by sharp teeth [5]. While in many countries teeth grinding is no longer allowed to be performed prophylactically and routinely, it is still performed as a remedy for lesions. Therefore, it is important to understand best practices for grinding to prevent welfare issues for those pigs that undergo the procedure.

There is still a lack of knowledge concerning the effects of different grinding methods or the complete abandonment of teeth grinding on the behaviour, health and welfare of piglets. Therefore, the aim of the present study was to compare three different treatments (no teeth grinding, teeth grinding using a standard rolling head, and teeth grinding using an alternative teacup head) concerning teeth injuries, behaviour, skin injuries, growth performance and mortality of suckling piglets.

## 2. Materials and Methods

### 2.1. Animals and Housing

The studies were conducted on a commercial pig farm in Northern Germany. At the time of the studies, about 1300 sows were kept on the farm (genetics: Danish Landrace × Yorkshire), and the sows were farrowed in a three-week rhythm. One farrowing group consisted of about 160 sows, and the suckling period was 28 days. There were 222 older farrowing pens in the barn with a 4.5 m^2^ pen area and 132 farrowing pens with an area of 5.5 m^2^. All pens were equipped with farrowing crates. Sows were moved to the farrowing compartment one week before the expected farrowing date. The farrowing pens were equipped with a heating plate and a red-light lamp for optimal temperature in the creep area of suckling piglets. The size of this creep area was 0.4 m^2^. Additionally, a trough, a nipple drinker and a cup system were available in each farrowing pen for the piglets. In the trough and the cup facility, electrolytes (“electrolyte mixture” B&K Agrar GmbH, Holdorf, Germany) were offered during the first two days of life. After that, the electrolytes were replaced by a milk replacer (“CulinaLiquid”, H. Bröring GmbH & Co. KG, Dinklage, Germany). From the third week of life, piglets were offered piglet feed (“CulinaFibre”) from the same company. The sows were fed twice daily during the first week of the piglets’ life and three times daily until the piglets were weaned with a farm-specific mixture (“SM LAK Landschwein Kr.”, H. Bröring GmbH & Co. KG, Dinklage, Germany). The creep area for piglets was littered with a lime mixture at birth, and a mat was placed behind the sows at the beginning of parturition. Farrowing was closely supervised by the farm staff. Litter equalisation was carried out within the first 24 h. As per zootechnical measures, the tails of all piglets were docked in the first 24 h of life, and the eight teeth were ground (canines and third incisor). In addition, the piglets were injected once with an iron preparation. On the third day of life, the males were castrated. Before castration, inhalation anaesthesia was applied using the PigletSnoozer device (GFS, Ascheberg, Germany). Isoflurane was used as an anaesthetic gas.

At first, a preliminary study was carried out. In the preliminary study, the effects of two different grinding methods on the occurrence of teeth injuries were investigated. In the following main study, behavioural and welfare aspects were investigated in piglets treated with the two grinding methods or not ground at all.

### 2.2. Preliminary Study

**Figure 1:**
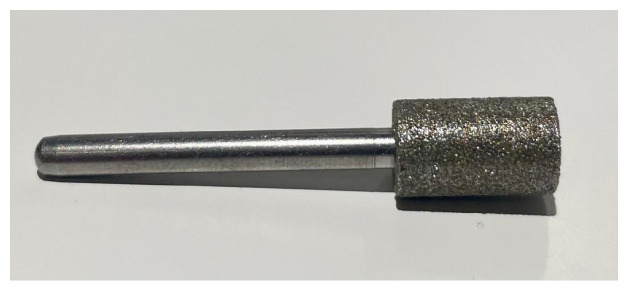
Roller-grinding head.

**Figure 2:**
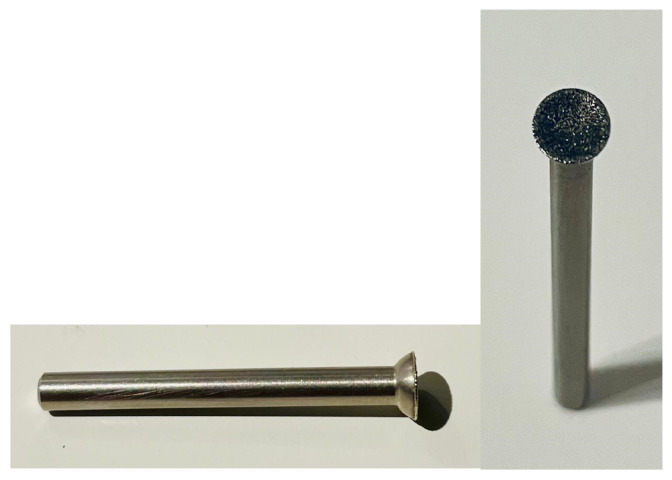
Teacup-grinding head.

The teeth of 200 randomly selected piglets originating from 24 litters were ground either with method RG (using the roller-grinding head, *n* = 100 piglets) or with method TCG (using the teacup-grinding head, *n* = 100 piglets). Teeth grinding was always performed by the same experienced person who had been carrying out this procedure on the farm for around 10 years. For method RG, the teeth of the piglets were ground in the first 24 h of life using an industrial drill grinder (IBS/E, Proxxon GmbH, Föhren, Germany) (Figure 1). This roller grinding head had a diameter of 7 mm and moved in rotation. For method TCG, an alternative grinding head (Figure 2) was used, which was produced by Wilofa Diamant, Fachbach, Germany, in accordance with an idea of Dr. Ute Hessling-Zeinen (Greven, Germany) [14]. The special feature of this grinding head is the concave grinding surface, which should be held perpendicular to the tooth during grinding, thus preventing slippage.

After teeth grinding, it was examined by two trained veterinarians whether the dental pulps of the canines and incisors in the upper and lower jaw were opened during grinding. Therefore, the animals were picked up individually and a dental probe was used to test each tooth to see whether the pulp had been opened or not.

### 2.3. Main Study

Four batches that were not part of the preliminary study were considered for the main study. The first, third and fourth batches comprised 24 sows each, and in the second batch, 25 sows were used. The sows were randomly selected; however, only sows up to the fourth litter (parity 4) were included in the study. Overall, almost 40% of the sows were gilts. The sows were randomly divided between older and newer farrowing pens in the barn. After farrowing, litter equalisation was carried out within the first 24 h, and the mean initial litter size was 16.28 ± 0.75 piglets. Only piglets with a minimum weight of >750 g were included in the study. All piglets of a litter were alternately assigned to one of the three treatment groups:

(1) grinding of the teeth with the roller-grinding head (method RG);
(2) grinding of the teeth with the teacup-grinding head (method TCG);
(3) control group, without teeth grinding (method CG).

Thus, one-third of littermates were treated with method RG, one-third with method TCG and one-third as CG. As in the preliminary study, teeth grinding was always carried out the day after birth by the same trained and experienced employee of the farm.

The piglets were always assigned to the respective treatment method in the same order. The order was always RG, TCG and then CG. The animals were always assigned to the treatments in descending order of weight, starting with the heaviest piglet. When allocation had been completed in the first litter, the heaviest piglet in the second litter was assigned to the following method. This ensured that the average body weight was approximately the same in each treatment group.

After the litters had been selected for the study, each sow received an individual sow card. Piglet losses, medical treatments and other abnormalities were recorded on this card. For piglet losses, it was accurately documented whether the piglet was a runt, was crushed or was non-viable. After birth, piglets were immediately included in the study. On the first day of life, they received a farm-specific ear tag with an individual additional number. The counterpart to the ear tag had a different colour. Each treatment method had its own colour: the RG method was red, the TCG method was yellow, and the CG was green. Thus, the groups could be observed in a differentiated manner.

#### 2.3.1. Lesion Score and Body Weight

Within the first 24 h postpartum, the piglets were individually weighed and examined for pathological changes or lesions on the head, ears, trunk, claws and limbs. A total of 1565 piglets were examined. Lesions were recorded using a score from 0 to 3 (Table 1).

Lesion scoring of piglets according to the above scheme was repeated once per week, always on the same day, until weaning on the 28th day of life. For piglet scoring, all animals of a litter were first removed from the pen and placed in a large cart. Each animal was then removed individually from this cart, clinically examined, and weighed. Since piglet scoring was repeated weekly, there were five examinations for each piglet in each litter. For further analysis, the scores of the head, ear and trunk were added up to a cumulated lesion score per piglet. The claw and limb parts of the body were largely neglected, as there were hardly any findings there.

#### 2.3.2. Behavioural Observations

Additionally, a behavioural observation of 650 piglets of 40 litters (a subset of the overall study group of 1656 piglets) was carried out as direct observation in the barn. For this purpose, an ethogram was first developed, which included the following behaviours: suckling, sitting, lying, standing, walking, food and water intake of piglets (Table 2).

In order to assess the behaviour of the individual animals in the litter, an animal-specific number was written on the back of the animals in conjunction with the first weighing within the first 24 h. This was performed using an approximately 2.5 mm wide brush and blue livestock marker spray. This marking allowed undisturbed observation of the animals from the aisle. The behavioural observation was always carried out by the same person. Within each litter, the behaviour of each individual piglet was observed and directly noted. When the behaviour of all littermates had been observed, the observer moved on to the next litter. In order to minimise the influence of the observing person on the animals’ behaviour, the observation started after a “habituation phase” of three minutes and the pen was not entered during that time.

The behaviour of the piglets was initially recorded before teeth grinding in two observations of each individual piglet. The litters were observed at intervals of about 30 min. After teeth grinding, a further eight observations were carried out for all animals. Again, the litters were analysed at intervals of about 30 min. Thus, each piglet was observed a total of 10 times within the first 24 h. On each of the following two days, five observations per piglet were made in the morning and five observations in the afternoon. The observations started at 07:30, half an hour after the sows had been fed. In the afternoon, the observation started at 14:00. The examination was repeated weekly for each litter in the same manner. The day after the lesion scoring and weighing was always selected for observation since the animal-specific back counts were rewritten on the day of lesion scoring. The behavioural analyses ended in the fourth week of life before weaning. Thus, a total of 60 observations were made per piglet.

### 2.4. Statistical Analysis

Statistical analysis was carried out using the program SAS Enterprise Guide and SAS software (version 7.14 and 9.4, respectively, Statistical Analysis Institute, Cary, NC, USA) for dental injuries, body weight and skin lesion score. The program R, version 4.3.1 [15] was used for the analysis of behaviour and mortality and some of the graphics. In all calculations, the significance level was set at *p* < 0.05.

For the statistical analysis of dental injuries (open pulps) per piglet, a logistic regression model was applied with the treatment (RG or TCG) as a fixed effect. Additionally, for analysis of pulp openings in the different teeth of the lower and upper jaw a mixed logistic model was used. The treatment (RG or TCG), jaw (lower or upper jaw), individual tooth and the interaction of tooth × jaw were set as the fixed effects, while the sow and the piglet (nested in sow) were considered as random effects.

For the analysis of lesion scoring, a cumulated lesion score was calculated for each individual piglet by summing up the individual scores of the body parts head, ear and trunk of an animal. The cumulated lesion score and the body weight were analysed by linear mixed models. The fixed effects were the treatment (RG, TCG or CG), sex of piglet, time (week), batch and the interaction treatment × time (week). The sow (nested in batch) was considered a random effect. Additionally, repeated measurements for the piglets (nested in sows) over time were incorporated using a heterogenous autoregressive correlation structure. To better fit the assumption of normality, the model for the lesion scores was fitted to the natural logarithm of the cumulated lesion scores. Tukey–Cramer adjustment was used for post hoc analysis.

For the statistical analysis of the behaviour and the mortality, mixed logistic regression models were used. For the analysis of the piglets’ behaviour, the following fixed effects were set: treatment (RG, TCG or CG), week, time of day (morning or afternoon), sex of piglets and the interaction treatment × week. The individual sow and piglet were considered random effects. Three models were applied using the behaviour “lying” as a reference:

Suckling vs. Lying
Feeding/drinking vs. Lying
Active behaviour (Walking/Standing/Sitting) vs. Lying

First of all, a global F-test was performed. If significant effects were found, pairwise comparisons using post hoc Wald tests were carried out. The *p*-values were adjusted using the Bonferroni method. For the statistical analysis of mortality, only data after teeth grinding were used. In the mixed logistic regression model, the following fixed effects were considered: treatment (RG, TCG or CG), individual body weight, litter size (i.e., the respective group size at the different time points after teeth grinding) and the interaction treatment × body weight. The individual sow and piglet were set as random effects. Global F-test and pairwise comparisons were performed as described above for behavioural analyses.

## 3. Results

### 3.1. Preliminary Study

**Figure 3:**
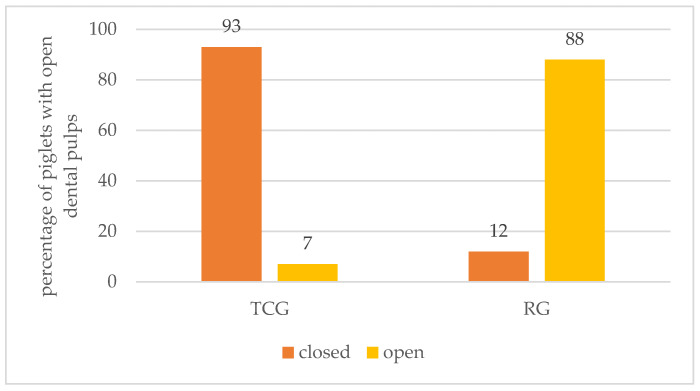
Percentage of piglets with open dental pulps (minimum: one open dental pulp per piglet) in the different treatment groups (RG = roller-grinding method and TCG = Teacup-grinding method).

A total of 1600 teeth from 200 piglets (100 piglets from the RG group and 100 piglets from the TCG group) were examined in the preliminary study. The probability of a piglet having a dental pulp opened was influenced by the treatment group, and RG piglets had a higher risk of an opened tooth than TCG piglets (*p* < 0.0001, odds ratio = 49.593, 95%—confidence interval = [24.3; 101.1]). Consequently, piglets in the TCG group had fewer opened dental pulps than piglets in the RG group (on average 0.08 ± 0.31 vs. 2.67 ± 1.67 opened dental pulps per piglet). When the TCG method was applied, most piglets had fully intact teeth. Piglets whose teeth were not completely intact had a maximum of two opened dental pulps. In the RG group, the majority of the piglets had at least one opened dental pulp (maximum: six opened dental pulps per piglet) (Figure 3). The RG method resulted in a total of 533 closed pulps and 267 open pulps, while the TCG method led to 792 closed pulps and only eight open pulps.

Furthermore, it was analysed descriptively which teeth in the upper or lower jaw were more likely to be opened with the different methods. With the RG method, in the upper jaw, the incisors (left incisor: 64%, right incisor: 44%, *n* = 100) were opened more frequently than the canines (left canine: 15%, right canine: 7%, *n* = 100). In the lower jaw, the canines were opened more frequently when using the RG method (left incisors: 16%, right incisor: 26%, left canine: 42%, right canine: 53%, *n* = 100).

**Figure 4:**
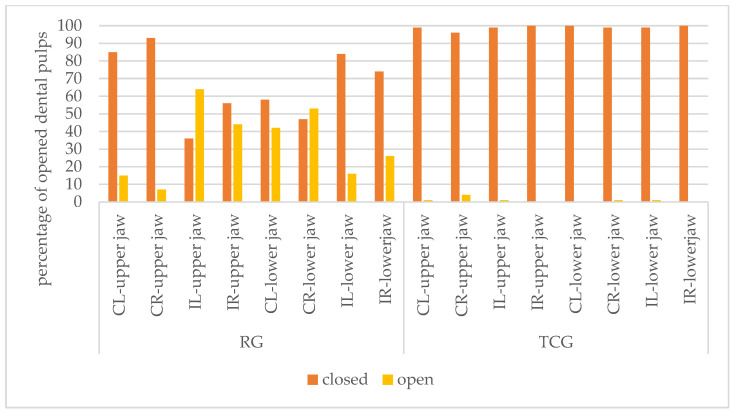
Percentage of opened dental pulps for the left canine (CL), right canine (CR), left incisor (IL) and right incisor (IR) for the upper and lower jaw of piglets when applying the RG (roller grinding) method and the TCG (Teacup-grinding) method.

When using the TCG method, the right canine was opened most frequently in the upper jaw (4%, *n* = 100). Of the left canines and incisors of the upper jaw, as well as the right canines of the lower jaw, only one was opened (1%, *n* = 100). Not a single dental pulp was opened in the right incisors of the upper jaw, the left canines of the lower jaw and the right incisors in the lower jaw (Figure 4). These findings could not be statistically tested because the corresponding model, including higher-order interactions, specifically group × jaw × tooth, showed quasi-complete separation, probably corresponding to the very small number of opened pulps in the TCG group. A significant effect of the interaction tooth × jaw (*p* < 0.0001) hinted at differences between teeth within and between the jaws.

### 3.2. Main Study

#### 3.2.1. Mortality

**Figure 5:**
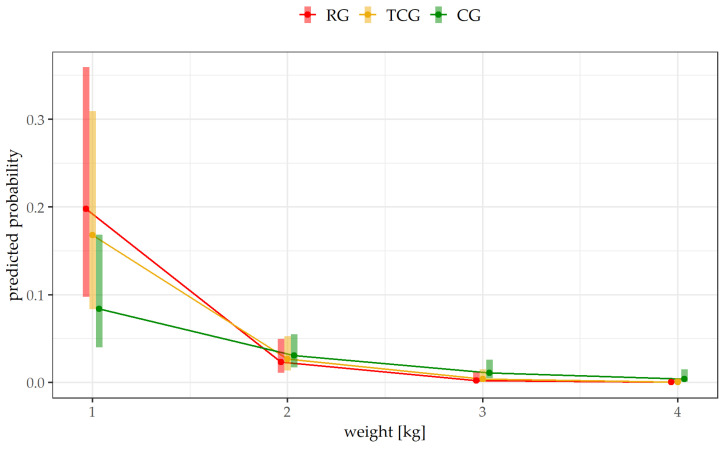
Predicted probability of piglets dying during the study depending on body weight in the different treatment groups (RG = roller-grinding method, TCG = Teacup-grinding method) and CG = control group). The bars indicate the confidence intervals. There were no significant differences between treatment groups (*p* > 0.05).

During the study, 8.3% (*n* = 54) of the total 650 piglets died. Regardless of their treatment group, the piglets that were included in this study only died from the second week of life onwards. The greatest number of deceased animals belonged to method RG (9.6%, *n* = 21 out of *n* = 217 piglets) and method TCG (9.1%, *n* = 20 out of *n* = 219 piglets). In CG, 6.1% (*n* = 13 out of *n* = 214 piglets) of piglets died. The results of the mixed logistic regression model revealed that the interaction between treatment and body weight was significant (*p* = 0.031). It was also shown that an increase in body weight of 1 kg resulted in a significantly higher probability of survival for the individual animal in all treatments (Table 3). Even if there was no significant difference (*p* > 0.05) in the probability of dying between the different treatments (RG, TCG and CG), it became evident that in a low weight range of 1 kg, there was a higher risk of mortality (predicted probability by the statistical model) in the RG and TCG groups, i.e., those groups where the teeth were ground compared to the control group without teeth grinding (Figure 5). From a statistical point of view, there was even a statistical tendency towards higher mortality in RG than in CG (*p* = 0.069).

#### 3.2.2. Body Weight and Lesion Score

The piglets in all three treatment groups had almost the same body weight at the beginning of the study (RG: 1.41 ± 0.31 kg, TCG: 1.42 ± 0.32 kg, CG: 1.42 ± 0.32 kg). The body weights were also very similar at the end of the study after the fourth week (RG: 7.28 ± 1.48 kg, TCG: 7.21 ± 1.50 kg, CG: 7.29 ± 1.51 kg). Further statistical analysis confirmed that there was no significant influence of treatment (*p* = 0.63) or of the interaction between treatment and week of the study on the body weights of piglets (*p* = 0.87). Thus, neither the method of teeth grinding nor the complete avoidance of teeth grinding had a statistically significant effect on the piglet weights.

**Figure 6:**
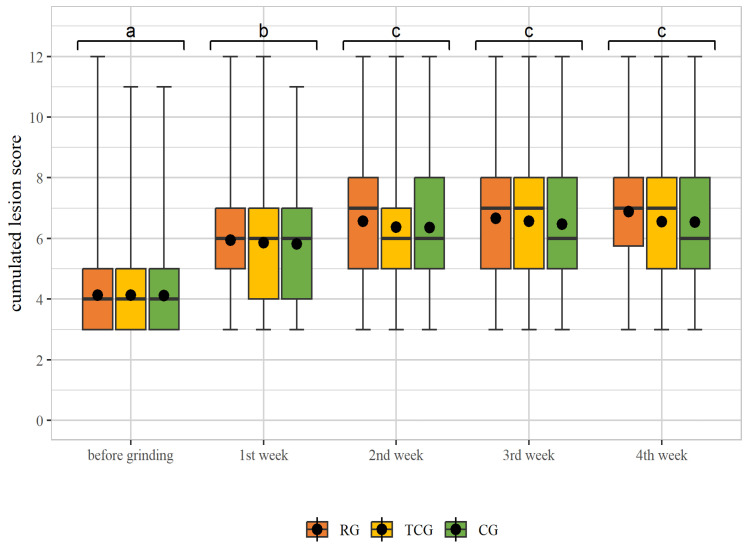
Boxplots (whisker from min to max) of cumulated lesion scores of the treatment groups (RG, TCG and CG) in different weeks of study. The group mean is drawn as a dot. Significant differences between the weeks (*p* < 0.05) are indicated by different letters. There were no significant differences between the treatment groups per week (*p* > 0.05).

The cumulated lesion score of piglets was not affected by the interaction of treatment and week of the study (*p* = 0.73). However, the cumulated lesion score was, in general, affected by the week of the study (*p* < 0.0001) and increased in all treatment groups over time from the beginning, i.e., before grinding (RG = 4.13 ± 1.41, TCG = 4.13 ± 1.36, CG = 4.11 ± 1.34) to the first week (RG = 5.94 ± 1.89, TCG = 5.86 ± 1.92, CG = 5.82 ± 1.86, *p* < 0.05) and to weaning in the fourth week of the study (RG = 6.89 ± 1.87, TCG = 6.56 ± 1.78, CG = 6.54 ± 1.79) (*p* < 0.05, Figure 6). There was no effect of the sex of the piglets on the cumulated lesion score (*p* = 0.447), while there was an effect of the batch (*p* < 0.0001).

#### 3.2.3. Behaviour

The results of the behavioural observations of the different treatment groups showed that lying was the most common behaviour of the piglets (60.5% of the observations per piglet), followed by suckling (31.1%). Active behaviour (running, standing, sitting) was observed less frequently (7.6%). The piglets were most rarely observed eating and drinking (0.8%). Three statistical models were applied (suckling, feeding/drinking and active behaviour) using the behaviour “lying” as a reference. The results revealed that there was no effect of the treatment group (RG, TCG or CG) on the analysed behaviours (*p* > 0.05). Detailed results for the behaviour of piglets are shown in Tables S1–S3 and in Figure S1 of Supplementary Materials.

## 4. Discussion

### 4.1. Tooth Injuries with Opening of the Dental Pulp

The results of the present study revealed that any form of teeth grinding can lead to the opening of the dental pulp. However, there were clear differences between the two grinding methods examined in the present study. While few open pulps were found for the TCG method, significantly more open pulps were detected when using the roller grinding head (CG). This result is in accordance with Ellert (2017), who found that the occurrence of pulp openings was significantly dependent on the shape of the grinding head, with the roller grinding head causing significantly more open dental pulps than the newly developed teacup grinding head [14]. In other previous studies, the surface of the teeth was also often not intact after teeth grinding [12,16]. Hay et al. showed that 40% of the dental pulps were opened after teeth resection by clipping the teeth [17]. In the present study, 88% of the piglets had at least one opened dental pulp using the RG method. With the TCG method, 7% of the piglets had opened dental pulps. This clearly contradicts the requirements set forth in EU Directive 2008/120/EC, which stipulates that a smooth and intact surface should be present when shortening teeth [7].

Considering the individual teeth, it was shown that the TCG method most frequently opened the canines in the upper jaw [14]. This was confirmed in the present study, where it was shown that the right canine of the upper jaw was opened most frequently when using the TCG method. Ellert (2017) justified this with the fact that canines in the upper jaw are easier to reach with the Teacup head than with the roller grinding head. As a result, the Teacup head may be used for a longer period of time and more tooth material is removed [14]. Nevertheless, it should be noted that a significantly lower number of pulps were opened with the TCG method compared to the RG method. The reason for this is the concave grinding surface, which adapts much better to the tooth surface.

In a study by Hessling-Zeinen (2014), the RG method was examined on four pig farms. It was shown that the incisors of the upper jaw were opened significantly more frequently than the other teeth [11]. This is also in accordance with the present study where it was shown that the left incisors (62%) and the right incisors (44%) of the upper jaw were opened most frequently. Hessling-Zeinen (2014) justified this with the fact that the crown tips of the incisors of the upper jaw anatomically protrude beyond the canines, and thus, the incisors have to be set significantly lower. This leads to a higher probability that the pulp cavity of the incisors will be opened. In the lower jaw, on the other hand, the canines are longer than the incisors, so the canines are more frequently affected than the incisors [11]. This assumption was confirmed in the present study. Based on the fact that the TCG method opened significantly fewer dental pulps than the RG method, it can be concluded that TCG is a more animal-friendly method when teeth resection is thought to be inevitable.

### 4.2. Mortality

In the present study, no significant difference was found in mortality between the three treatment groups. However, in a low weight range of 1 kg, there was a tendency towards higher mortality in RG than in CG. This may be explained by the high number of opened dental pulps in RG being an entry point for pathogens. The open pulps can cause further pathological changes in the pulp and periodontium, which, in the worst case, can result in osteomyelitis. The consequences are chronic pain that can be accompanied by a reluctance to eat [18], resulting in further health issues. Previous studies on mortality have only compared intact, ground and clipped teeth. There are studies where no influence of the treatment method on the mortality risk has been determined [16,19]. This contrasts with the study by Hansson and Lundeheim (2012), which found a significantly higher mortality rate in piglets with non-ground teeth [20]. It is important to note that only a few piglets died during the present study. Thus, the results relate to a small sample size. With a larger number of deceased piglets, a statistically detectable difference between the treatments might have become apparent, at least in a low weight range. Further studies on more farms with larger numbers of animals are therefore recommended in order to determine the risk of illness and increased mortality due to teeth grinding. In the present study, the animals only died from the second week of life onwards. Before grinding and in the first week, no piglet used for the present study died. Many studies have already shown that the piglets’ chance of survival is heavily dependent on their birth weight. Lighter animals have less energy stores and immature immune systems and are therefore more susceptible to hypothermia, starvation or crushing by the sow [21,22,23,24]. A study by Chris et al. (2012) showed that piglets with a birth weight of <700 g only had a 30% chance of survival, whereas animals weighing > 1.8 kg had a 90% chance [25]. For this reason, only animals with a birth weight > 750 g were included in the present study. This may have resulted in mortality remaining low in this study, with deaths not occurring until 2 weeks after the start of the study. Furthermore, the treatments CG, TCG and RG were equally divided among the animals of different weights. Nevertheless, this study also proved that the less weight an animal had, the higher the probability of death.

### 4.3. Lesion Score

In the present study, there were no differences in the cumulated lesion scores between the groups with teeth grinding and the control group without teeth grinding. Similar results were reported by Gallois et al. (2005), who found that only 10% of the piglets that were not ground showed deep bite wounds in the head area [16]. This raises the question of whether a high number of dental injuries caused by teeth grinding should be accepted in order to protect the piglets from bite injuries. In a survey conducted by Chou et al. (2022), it was shown that leaving the teeth of piglets intact is considered the main cause of the lesions that occur in suckling piglets. For this reason, teeth grinding in piglets is a remedy to protect them from lesions. According to the survey conducted among farmers, teeth grinding is considered the most sensible preventive measure to reduce pathological changes, especially in the facial area of piglets [5]. Several studies have shown that lesions occurred more frequently when piglets had untreated, intact teeth compared to shortened teeth [9,12,16,26,27]. However, this was not confirmed in the present study. In the present study, there was a significant increase in the lesion score from birth to the second week of life, regardless of whether the teeth were ground or not. It is possible that further rank-order fights caused more injuries during this period. It also has to be considered that in the present study, injuries at several body parts were determined, and possibly the pen equipment such as the hard slatted floor led to injuries as well.

### 4.4. Body Weight

The relationship between weight gain and lesions of piglets is not clear in the scientific literature. For instance, it was found that facial lesions did not lead to lower weight gain in piglets with intact teeth [4,9,28,29,30,31]. In the present study, the lesion score of piglets was not influenced by the treatment TCG, RG or control, nor was the body weight gain. Earlier studies regarding the body weight gain of suckling piglets after teeth grinding showed contradictory results. In some previous studies, piglets that underwent tooth resection showed a significant reduction in weight gain compared to non-ground piglets [32,33], whereas, in other studies, no influence of teeth grinding on weight gain was found [16,27]. Only Hutter (1993) was able to show that weight gains were higher in ground piglets than in piglets with clipped and non-clipped teeth [12]. A study by Holyoake et al. (2004) even showed that the weaning weights of piglets with clipped teeth were higher than those with ground teeth. However, the highest weights were found in piglets whose teeth had not been ground [26]. Fraser and Thompson (1991) reported on so-called selective grinding. There, only some of the piglets in a litter were ground, and it was shown that the piglets without ground teeth were more likely to be found at the sow’s high-yielding front teats, and the piglets with ground teeth were more likely to be left behind [33]. The teat positions of piglets were not analysed in the present study. However, selective grinding was carried out in each litter as well since the piglets of the control group were in the same litter as the piglets of the two other treatments. Nevertheless, no differences in weight development were found between littermates with and without teeth grinding.

### 4.5. Behaviour

The influence of teeth grinding on piglet behaviour has already been investigated in several studies. Bataille et al. (2002) found a change in behaviour when comparing four different treatment groups: piglets with ground teeth, clipped teeth, a simulation of grinding and piglets with intact teeth. Especially during grinding the piglets showed a clearly increased activity of the hind limbs [30]. An increased vocalisation with very loud screams was determined as well [34,35]. The animals with ground teeth showed significantly higher activity on the udder and also walked more overall after grinding than the non-ground animals [36]. These results may be a confirmation of Noonan’s statement that pain leads to increased activity. This was justified by the release of endorphins through suckling, which has an analgesic effect [37], and it was supported by an observation of Hay et al. (2004), who also found that pain led to increased restlessness [17]. According to Boyle et al. (2002), the explorative behaviour of piglets without teeth grinding was higher in the first 15 days than that of the treated piglets, showing more sleeping behaviour. However, an increased running activity of the untreated piglets was not detected [38]. In the present behavioural analysis, no difference in behaviour was found between the three treatment methods. This largely contradicts previous studies. Nonetheless, a difference to previous studies is the fact that in the present study the animals from all three treatment groups were represented in the same litter. One should bear in mind that the behaviour of some piglets may influence the behaviour of the other piglets within the same litter, and therefore, differences in behaviour between treatments may not have been evident in this study design.

## 5. Conclusions

The present study showed that grinding piglet teeth with the teacup grinding head is a more animal-friendly alternative to the frequently used roller grinding head. However, in principle, each of the two grinding methods can open the dental pulp and thus lead to pain and possible subsequent damage. In this study, there were no differences in mortality, weight gain, skin lesions and behaviour of piglets between the experimental groups and the untreated control group. This highlights the importance of carefully considering the need for teeth grinding for each individual farm. If it is not possible to avoid teeth grinding on a farm, it is recommended to switch to the teacup grinding head in the interests of animal welfare.

## Figures and Tables

**Table 1:** Skin lesion scoring system for piglets.

Score	Definition
0	no injuries
1	small number (<5) of superficial ^1^ scratches
2	medium number of superficial scratches (5–10) or a small number (<5) of deep ^2^ scratches
3	high number of superficial scratches (>10) or medium to high number of deep scratches (>5)

^1^ superficial: injuries to the upper layers of the skin, slight reddening, minimal bleeding/scab formation. ^2^ deep: injuries to deeper skin layers with redness and bleeding/scab formation, possibly necrotic/purulent.

**Table 2:** Ethogram of the analysed behaviours of piglets.

Behaviour	Definition
lying	The piglet’s body is not held up by the four legs.
suckling	The piglet has direct oral contact with the sow’s teat.
active behaviour	
running	The piglet shows any form of locomotion.
standing	The piglet stands on all four legs.
sitting	The piglet relieves the hind limbs; the main part of the body weight rests on the forelimbs, which are stretched out.
eating/drinking	The piglet’s snout is in the feed bowl, and the piglet is eating, or its snout is in the water trough.

**Table 3:** Results of the mixed logistic regression model for mortality of piglets relating to body weights and treatment groups calculating the factor (odds ratio) by which the probability of dying is higher if an animal is 1 kg lighter than a reference animal (x/x + 1 kg); CG = control group without teeth grinding, TCG = teacup-grinding, RG = roller-grinding.

Method	Weight (kg)	Odds Ratio	SE	*p*-Value	Confidence Interval (2.5%)	Confidence Interval (97.5%)
CG	x/x + 1 kg	2.876	0.854	<0.001	1.413	5.859
TCG	x/x + 1 kg	7.291	3.308	<0.001	2.461	21.605
RG	x/x + 1 kg	10.348	5.348	<0.001	3.003	35.660

## Data Availability

The data presented in this study are available on reasonable request from the corresponding author. The data are not publicly available due to privacy.

## Supplementary Materials

The following supporting information can be downloaded at: https://www.mdpi.com/article/10.3390/ani14091318/s1, Table S1: Structured table for the mixed logistic regression model, including the table of coefficients as well as the results of the F-test for the behaviour “suckling” vs. “lying”; Table S2: Structured table for the mixed logistic regression model including the table of coefficients as well as the results of the F-test for the behaviour “eating/drinking” vs. “lying”; Table S3: Structured table for the mixed logistic regression model, including the table of coefficients as well as the results of the F-test for the “active behaviour” vs. “lying”; Figure S1: Mean percentage for different behaviours ((a) lying, (b) suckling, (c) eating/drinking, (d) active behaviour) related to all observations per piglet in the different treatment groups (RG = roller-grinding, TCG = teacup-grinding, CG = control group without teeth grinding) and in different weeks of the study (before grinding until the fourth week after grinding).
