# Supplementary material for: Effects of Two Different Methods of Teeth Grinding on Dental Injuries, Skin Lesions, Growth and Behaviour of Suckling Piglets Compared to a Non-Treated Control Group
Source: Animals (Basel). 2024 Apr 28;14(9):1318. doi: 10.3390/ani14091318 (PMC11082941; doi:10.3390/ani14091318)
Supplement: Supplementary file 1 [file animals-14-01318-s001.zip › animals-2951759-supplementary.pdf]

Table S1. Structured table for the mixed logistic regression model, including the table of coefficients as well as the results of the F-test for the behaviour "suckling" vs. "lying".

	Estimate	Odds Ratio	Std. Error	df1	df2	f-Value	*p*-Value
intercept	0.121		0.112				
method = TCG	0.024	1.024	0.140				
method = RG	0.045	1.046	0.140				
time = 1. week	−0.701	0.496	0.103				
time = 2. week	−1.003	0.367	0.112				
time = 3. week	−0.840	0.432	0.113				
time = 4. week	−1.205	0.300	0.115				
morning/afternoon = afternoon	−0.067	0.935	0.024				
sex = male	0.024	1.024	0.024				
method = TCG; time = 1. Week	−0.012	0.988	0.145				
method = RG; time = 1. week	−0.057	0.945	0.146				
method = TCG; time = 2. Week	−0.005	0.995	0.158				
method = RG; time = 2. Week	0.085	1.089	0.159				
method = TCG; time = 3. Week	−0.034	0.967	0.159				
method = RG; time = 3. Week	−0.102	0.903	0.160				
method = TCG; time = 4. Week	0.019	1.020	0.162				
method = RG; time = 4. week	0.110	1.117	0.163				
model term							
method				2	∞	0.886	0.412
time				4	∞	91.230	<0.0001
morning/afternoon				1	∞	7.660	0.006
sex				1	∞	0.948	0.330
method × time				8	∞	0.945	0.477

Table S2. Structured table for the mixed logistic regression model including the table of coefficients as well as the results of the F-test for the behaviour "eating/drinking" vs. "lying".

	Estimate	Odds Ratio	Std. Error	df1	df2	f-Value	*p*-Value
intercept	−5.833		0.931				
method = TCG	0.629	1.876	1.131				
method = RG	0.866	2.378	1.123				
time = 1. week	0.905	2.473	0.928				
time = 2. week	0.498	1.645	0.980				
time = 3. week	1.324	3.759	0.947				
time = 4. week	1.988	7.299	0.930				

morning/afternoon = afternoon	−0.737	0.478	0.123				
sex = male	0.073	1.076	0.131				
method = TCG; time = 1. Week	−0.817	0.442	1.154				
method = RG; time = 1. week	−1.161	0.313	1.148				
method = TCG; time = 2. Week	−0.572	0.564	1.230				
method = RG; time = 2. Week	−0.326	0.722	1.210				
method = TCG; time = 3. Week	−0.416	0.660	1.181				
method = RG; time = 3. Week	−0.503	0.605	1.170				
method = TCG; time = 4. Week	−0.369	0.692	1.153				
method = RG; time = 4. week	−0.567	0.567	1.146				
model term							
method				2	∞	0.869	0.419
time				4	∞	30.421	<0.0001
morning/afternoon				1	∞	35.911	<0.0001
sex				1	∞	0.314	0.575
method × time				8	∞	0.725	0.669

Table S3. Structured table for the mixed logistic regression model, including the table of coefficients as well as the results of the F-test for the "active behaviour" vs. "lying".

	**Estimate**	**Odds Ratio**	**Std. Error**	**df1**	**df2**	**f-Value**	***p*-Value**
intercept	−3.327		0.931				
method = TCG	0.705	2.024	0.451				
method = RG	0.962	2.618	0.437				
time = 1. week	1.060	2.885	0.370				
time = 2. week	1.492	4.448	0.374				
time = 3. week	1.745	5.727	0.373				
time = 4. week	1.563	4.773	0.374				
morning/afternoon = afternoon	−0.207	0.813	0.041				
sex = male	−0.084	0.919	0.042				
method = TCG; time = 1. Week	−0.681	0.506	0.458				
method = RG; time = 1. week	−1.052	0.349	0.445				
method = TCG; time = 2. Week	−0.793	0.452	0.465				
method = RG; time = 2. Week	−0.916	0.400	0.452				
method = TCG; time = 3. Week	−0.703	0.495	0.463				
method = RG; time = 3. Week	−1.056	0.348	0.451				
method = TCG; time = 4. Week	−0.725	0.484	0.465				
method = RG; time = 4. week	−0.902	0.406	0.452				
model term							
method				2	∞	1.666	0.189
time				4	∞	52.300	<0.0001
morning/afternoon				1	∞	25.265	<0.0001
sex				1	∞	4.048	0.044
method × time				8	∞	1.219	0.283

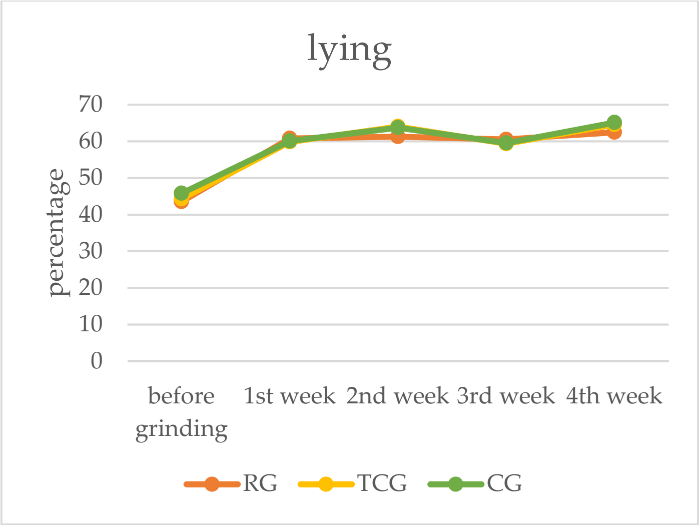
lying
70
60
50
40
30
20
10
0
percentage
before grinding
1st week
2nd week
3rd week
4th week
RG
TCG
CG

(**a**)

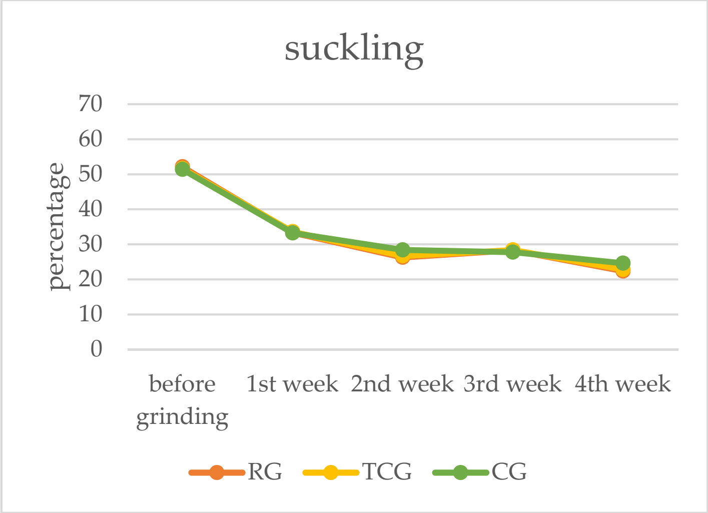
suckling
70
60
50
40
30
20
10
0
percentage
before grinding
1st week
2nd week
3rd week
4th week
RG
TCG
CG

(**b**)

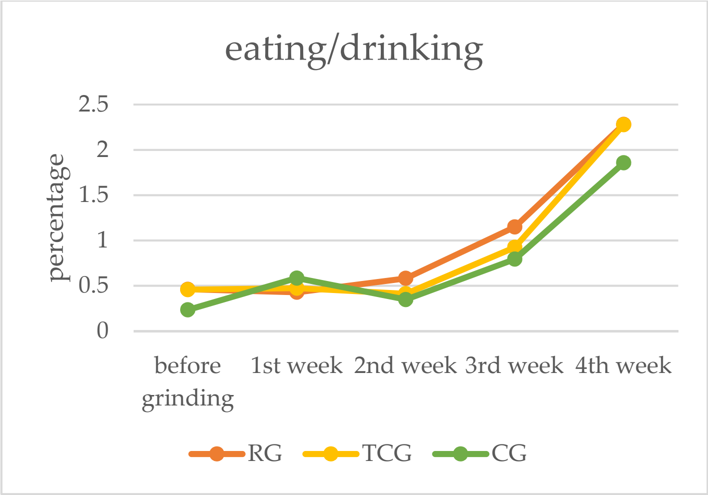
eating/drinking
2.5
2
1.5
1
0.5
0
percentage
before grinding
1st week
2nd week
3rd week
4th week
RG
TCG
CG

(**c**)

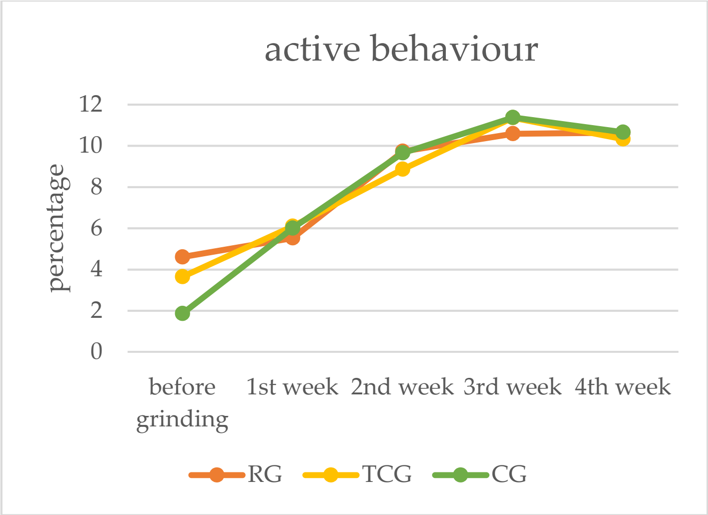

(**d**)

Figure S1. Mean percentage for different behaviours ((**a**) lying, (**b**) suckling, (**c**) eating/drinking, (**d**) active behaviour) related to all observations per piglet in the different treatment groups (RG = roller-grinding, TCG = teacup-grinding, CG = control group without teeth grinding) and in different weeks of the study (before grinding until the fourth week after grinding).
